# Supplementary material for: Long-range optofluidic control with plasmon heating
Source: Nat Commun. 2021 Mar 31;12:2001. doi: 10.1038/s41467-021-22280-3 (PMC8012589; doi:10.1038/s41467-021-22280-3)
Supplement: Supplementary file 1 — Supplementary Information [file 41467_2021_22280_MOESM1_ESM.pdf]

SUPPLEMENTARY INFORMATION

Long-range optofluidic control with plasmon heating

B. Ciraulo,[1,2] J. Garcia-Guirado,[1,2] I. de Miguel,[1] J. Ortega Arroyo,[1,2]* R. Quidant[1,2,3]*

AFFILIATIONS

[1] ICFO – Institut de Ciències Fotòniques, The Barcelona Institute of Science and Technology, 08860 Castelldefels (Barcelona), Spain.

[2] Nanophotonic Systems Laboratory, Department of Mechanical and Process Engineering, ETH Zurich, 8092 Zurich, Switzerland.

[3] Institució Catalana de Recerca i Estudis Avançats (ICREA), 08010 Barcelona, Spain.

*Corresponding authors. Email: jarroyo@ethz.ch, rquidant@ethz.ch

Supplementary Figures

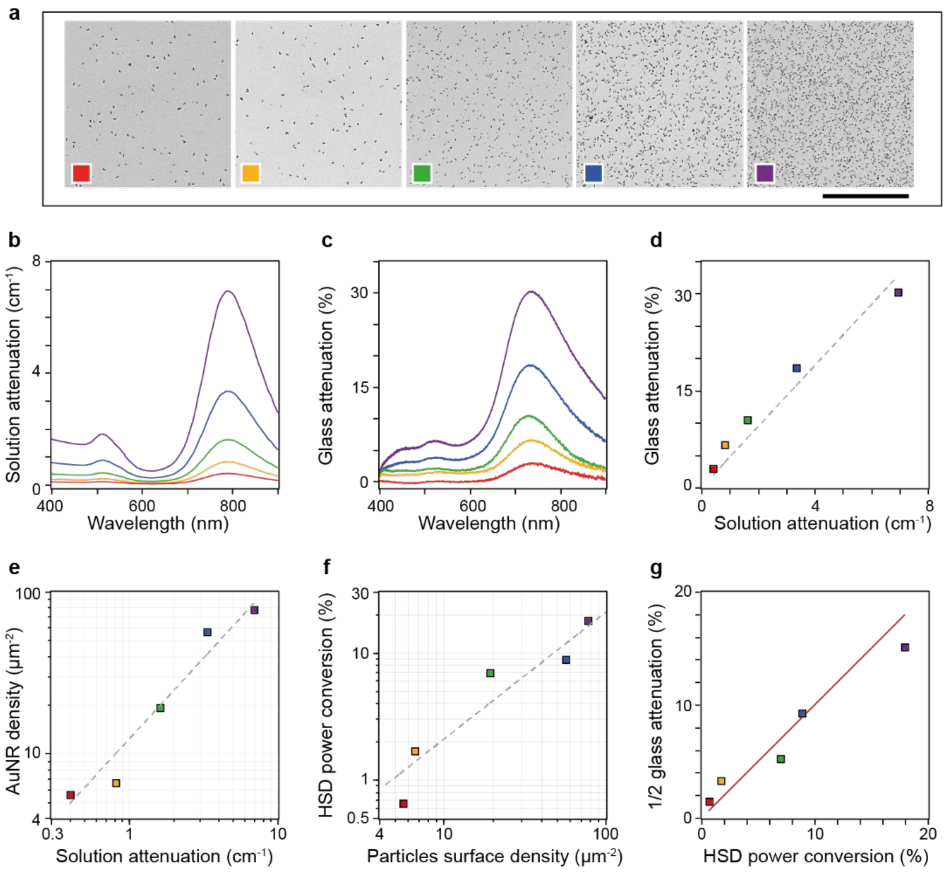

Supplementary Figure 1. AuNR and glass substrate characterization. **(a)** Scanning electron microscope images of functionalized glasses prepared using solutions with different AuNR concentrations **(b)** Solution attenuation and **(c)** corresponding functionalized glass attenuation. **(d)** Maximum glass substrate attenuation vs maximum solution attenuation. **(e)** AuNR surface density on the glass substrate as a function of different starting AuNR concentration used for the coating. **(f)** Light to heat power conversion as a function of AuNR surface density determined from the HSD. **(g)** Comparison between heat conversion efficiency as retrieved from the HSD and from the glass attenuation spectra, with the red line indicating a one-to-one correspondence. Color encodes each different AuNR solution. Scale bar: 5 μm. Dashed lines correspond to linear fits to the data.

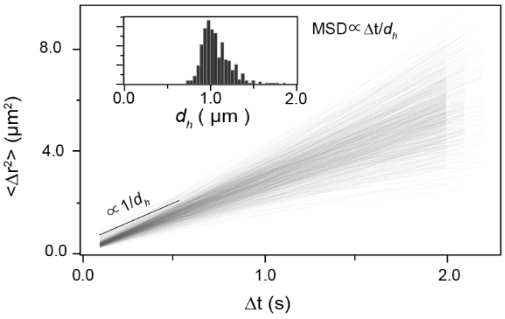

Supplementary Figure 2. Tracer particle characterization. Mean-squared-displacement curves for each tracer particle acquired under equilibrium conditions. The diffusion coefficient and specifically the hydrodynamic diameter, d_h, is determined from the slope of each curve considering lag times up to 0.5 s. Inset: Obtained hydrodynamic particle size distribution.

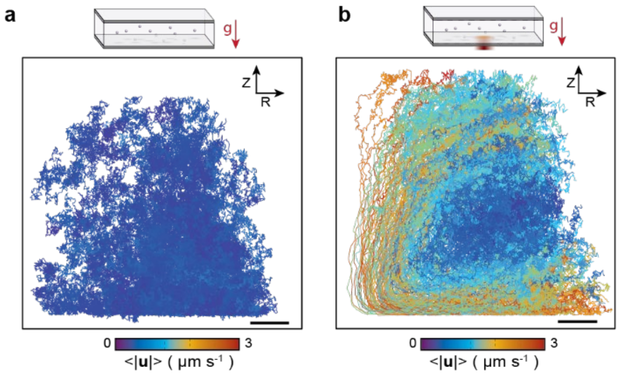

Supplementary Figure 3. Radial projection of 3D tracks for the perpendicular sample orientation. **(a, b)** Spatial distribution maps in the collapsed RZ plane of tracer particle trajectories under thermal equilibrium and non-equilibrium conditions reported in Figure 2c and 2d in the main text, respectively. Scale bars: 10 μm.

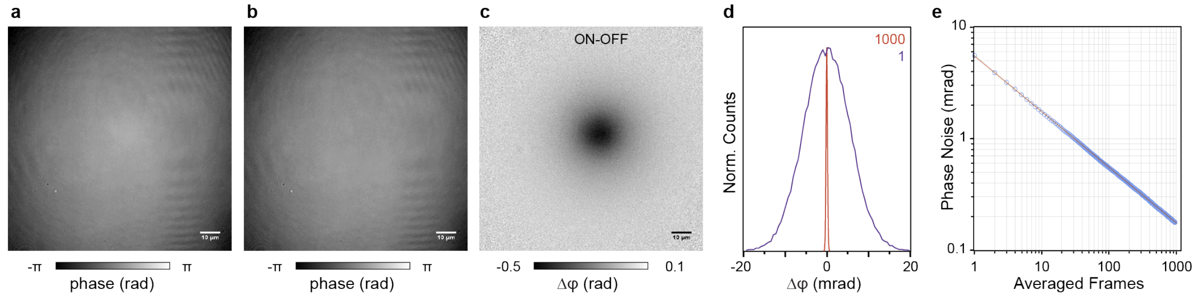

Supplementary Figure 4. Pump-probe phase imaging and sensitivity. **(a, b)** Phase image recorded with the pump On and pump Off, respectively. **(c)** Phase difference, pump On - pump Off, attributed to a steady-state temperature increase in the sample. **(d)** Phase noise distribution of a single (blue) and after averaging 1000 (orange) pump On- pump Off images, respectively. **(e)** Phase noise as a function of number of differential images averaged, with a line indicating shot noise limited behaviour. Scale bars: 10 µm.

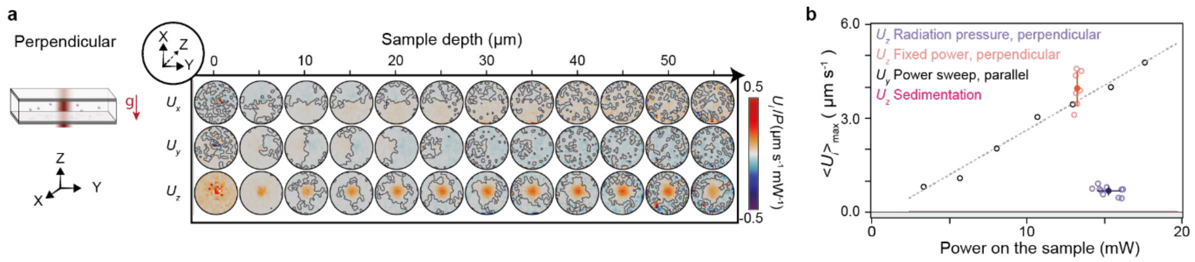

Supplementary Figure 5. Radiation pressure and sedimentation contributions. **(a)** Schematic of the sample orientation (left panel) and corresponding 3D velocity field map capturing the contributions attributed solely to radiation pressure (right panel). Color encodes the flow velocity normalized by the power of the pump beam on the sample. **(b)** Maximum average flow velocity along the indicated vector coordinate as a function of pump power impinging directly on the sample. The average flow velocity is determined from an area of 20 x 20 µm^2 around the centre of the pump beam. The radiation pressure contribution is obtained from a blank flow cell, i.e. without AuNR immobilized on the substrate as pictured in **(a)**. The sedimentation contribution is negligible and falls below our measurement uncertainty, therefore we report the theoretical values for a 1.0 µm polystyrene particle. Each empty circle represents a different experiment and the solid circles represent average values. Errors bars indicate one standard deviation distance away from the mean of the data. Dashed line represents a fit to the power sweep dataset.

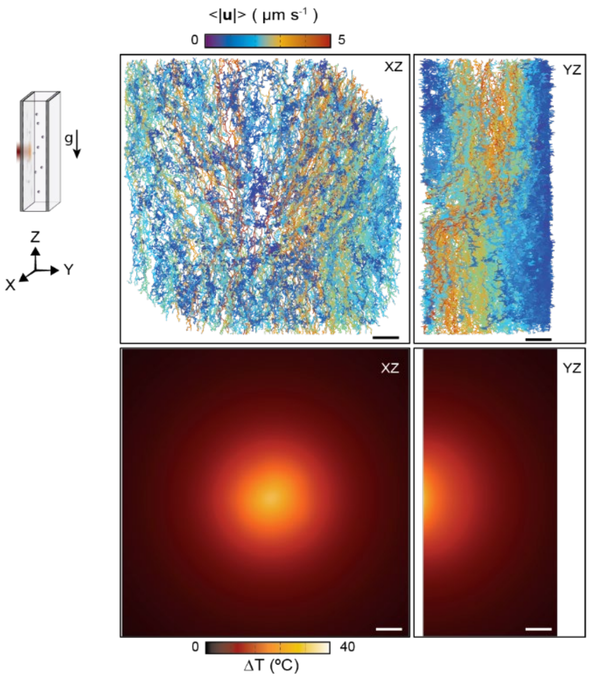

Supplementary Figure 6. Correlative 3D single particle tracking and thermometry for parallel sample orientation. Schematic depicting the orientation of the optofluidic platform with respect to gravity. Top: Spatial distribution map in the XZ (left) and YZ (right) planes of the trajectories of each tracer particle under thermal non equilibrium conditions. Bottom: corresponding experimentally measured 3D steady-state temperature distribution. Each track is assigned a color according to the average of the magnitude of the instantaneous velocity. Scale bars: 10 µm.

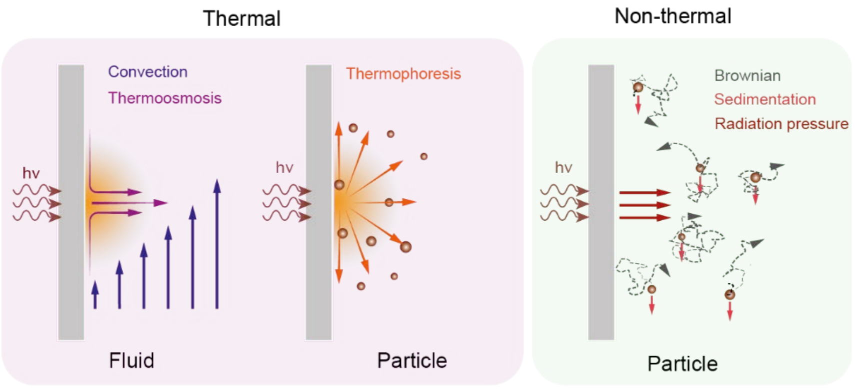

Supplementary Figure 7. Contributing phenomena to the dynamics of the parallel orientation. Schematic representation of the different thermal and non-thermal induced phenomena responsible for the dynamics observed in the parallel orientation system. Arrows indicate direction of motion attributed to each contribution.

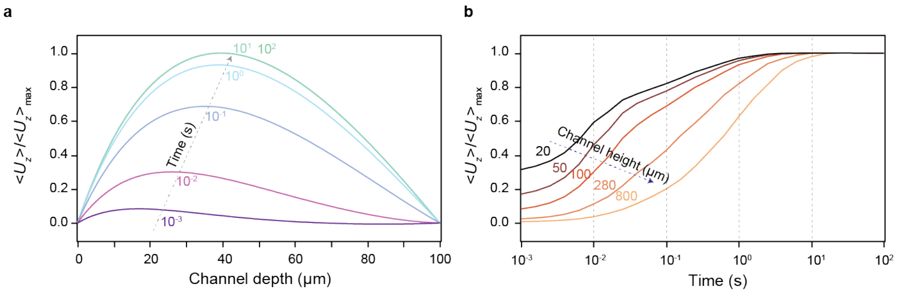

Supplementary Figure 8. Simulated temporal evolution of the convection-driven flow. **a)** Normalized flow profile due to convection driven dynamics as a function of time for a channel height of 100 µm with a parallel orientation and heat source size of 20 µm. **b)** Normalized temporal evolution of convective flow as a function of time for different channel heights.

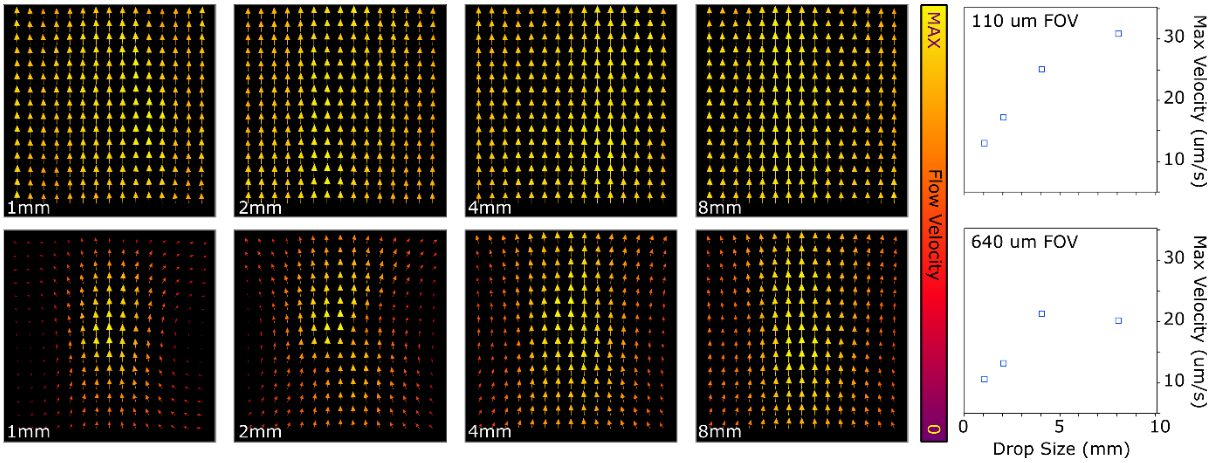

Supplementary Figure 9. Effects of boundaries on convective flow. Left: Flow velocity maps obtained at the center of different sized water droplets as seen from a 110 x 110 µm^2 FOV (top panel) and 640 x 640 µm^2 FOV (bottom panel). The flow velocity maps are computed by particle imaging velocimetry analogous to Fig. 6 in the main text. A maximum temperature increase of 70 ºC is achieved for all measurements by illuminating the centre of the FOV with a 20 µm pump beam. Right: Corresponding maximum flow velocity as a function of drop size.

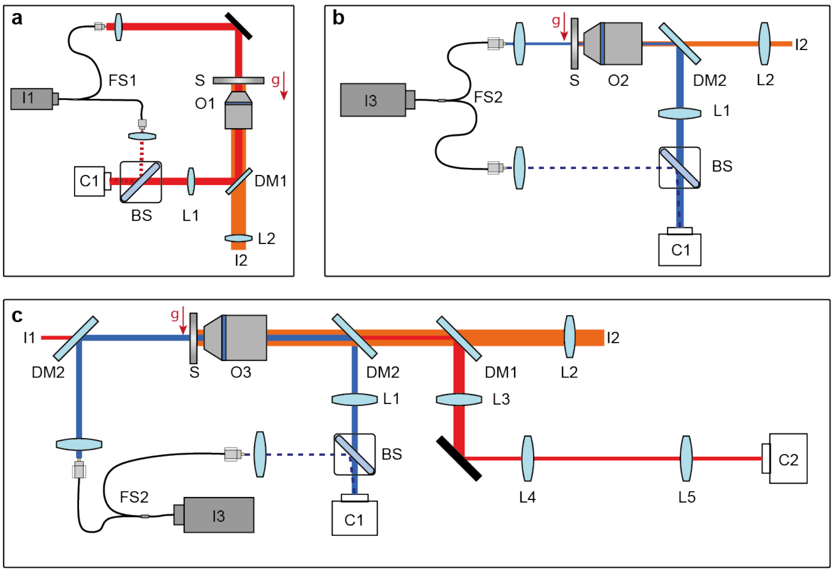

Supplementary Figure 10. Optical setup scheme. **(a)** Schematic diagram for the setup in which the sample is oriented perpendicularly with respect to gravity. **(b)** Same as **(a)** but for the sample oriented parallel with respect to gravity. **(c)** Modification of setup shown in **(b)** to visualize the sample with two different magnifications. **BS:** 90:10 beam splitter (Thorlabs BSX16), **C1:** CMOS camera (Basler acA1920-155um), **C2:** CMOS camera (Pixelink D755CU), **DM1:** 650nm long pass dichroic mirror (Thorlabs DMLP650L), **DM2:** 490 nm long pass dichroic mirror (Thorlabs DMLP490L), **FS1**: 1x2 optical fiber splitter (Thorlabs TN632R5F1), **FS2:** 1x2 optical fiber splitter (Thorlabs TW470R5F1), **I1:** 635 nm diode laser (Lasertack LDM-465-3000-c), **I2:** 780 nm diode laser (Lasertack LDM-780-200-c), **I3:** 465 nm laser diode (Lasertack LDM-465-3000-c), **L1:** 250 mm lens (Thorlabs AC508-250-A), **L2:** 500 mm focal length lens (Thorlabs AC254-500-B), **L3:** 150 mm lens (Thorlabs AC508-150-A), **L4:** 300 mm lens (AC508-300-A), **L5:** 100 mm lens (AC508-100-A), **O1:** Olympus 40x/0.65 NA (PLANFL40X), **O2:** Olympus 40x/0.75NA (UPLFLN40XPH), **O3:** Olympus 20x/0.40NA (PLN20XPH), **S:** Sample .

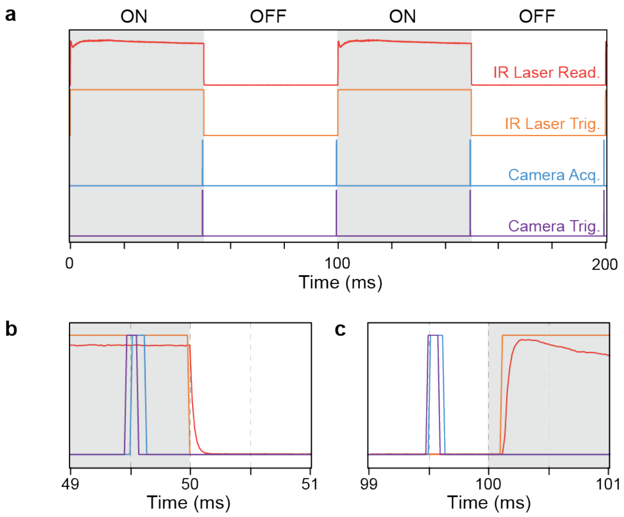

Supplementary Figure 11. Pump-probe synchronization scheme. **(a)** Representative experimental synchronization pulses for a pump-probe thermometry assay. The probe laser is triggered synchronously with the camera acquisition and is omitted for simplicity. **(b)** Zoomed section for recording of a pump ON frame. **(c)** Zoomed section for recording of a pump OFF frame. Trigger pulse frequency: 20 Hz, camera exposure time: 100 µs, pump pulse duration: 49.9 ms.

SUPPLEMENTARY NOTES

Supplementary Note 1: Differences between locally and uniformly heated systems:

Focussing on the model flow cell system presented in our work, the first key difference between locally and uniformly heated systems is the resulting temperature field in the water layer, which determines the resulting fluid and particle dynamics. Here, a critical observation is that heat diffuses through the surrounding water and glass sidewalls and dissipates to the environment (air) by natural convection from the glass surfaces.

In the case of a locally heated system, despite the low heat exchange rate of the external air convection, it is possible to reach a steady state temperature distribution thanks to the relatively large size of the thermal bath (surrounding glass and water) compared to the heat source. Thus, at steady-state, the temperature T at any point at distance r from the heat source at temperature T_o, is well approximated as $T=T_0/r$ (with r much greater than the size of the heat source). In the case of a uniformly heated system[1,2], there is no such additional thermal bath within the flow cell. Therefore, to obtain a steady state temperature field in the water layer, the system requires a heat sink on the opposing sidewall, which can be achieved by either using a material with a very high thermal conductivity[3] (silicon, sapphire), and/or keeping the surface at a fixed temperature via active cooling. The resulting temperature field will be linear, with the distance from the heated surface all across the sample, i.e. proportional to r.

A second key difference is the nature of flow expected in the case of uniformly heated surfaces. In the parallel orientation, the fluid flows upwards close to the hot surface, and downwards close to the cold surface. In the perpendicular orientation, specifically when the system is heated from the bottom, convection arises from an instability when the Rayleigh number exceeds a critical value[4]. This type of convection goes by the name of Reynard-Bénard convection, and leads to the appearance of local convection cells located throughout the sample. Both these scenarios are very different from locally heated systems, such as those presented in the work.

A third key difference involves thermal inertia of the system, which determines the cooling and heating dynamics, which in turn affects the particle and fluid dynamics. Namely, for locally heated systems, the thermal inertia is small, leading to faster heating and cooling dynamics, which result in a faster system response. This translates into greater reconfigurability and finer control compared to uniformly heated ones. As shown in Supplementary Figure 8, the onset of convection-based flow takes place at most within the first 10 s after heating

A fourth key difference is the contribution from thermophoresis and thermo-osmosis, which determine whether particles and fluid move along or against the thermal gradient[5]. These transport mechanisms depend on the temperature gradients, which in the case of locally heated systems are localized around the heat source and tend to be greater compared to the uniformly heated system. These dynamics are evident close to the heat source as shown in the spatial maps in Figure 3c of the main text.

To conclude, although locally and uniformly heated systems can achieve similar long-range fluid actuation, the underlying temperature field distribution, fluid transport mechanisms microscale dynamics, and conditions that lead to such behaviour are very different.

SUPPLEMENATARY METHODS

AuNR Solution and glass substrate characterization

The characterization of the AuNR solutions and of the glass substrate coating process was conducted through optical absorbance and scanning electron microscopy (SEM). Several glasses were prepared using different AuNR concentration solutions and the density of particles immobilized on the surface was determined from the SEM images (Supplementary Figure 1a). The concentration of the AuNR solutions was characterized by their attenuation spectra (Supplementary Figure 1b), where typical transversal and longitudinal plasmon resonance bands at around 520 nm and 780 nm respectively are clearly visible. The nanorods of this size are ideal in our application because of their absorption peak around 780 nm, which matches the pump laser, and low absorbance at the probe laser wavelengths of 465 nm and 635 nm. The attenuation value of the coated glasses was measured using a microscope-coupled spectrometer (Fig. S1c).

Specifically, the glass attenuation is defined as the percentage reduction in light transmission caused by the immobilized gold layer and is determined by measuring the transmission spectra of a glass with uniform AuNR coating on both sides and that of a plain glass. Since the transmission spectra are measured for a dry glass in air, the resonance frequencies are significantly blue shifted with respect to the AuNR solution due to the changes in refractive index of the surrounding medium. The glass maximum attenuation scales linearly with the solution maximum attenuation (Supplementary Figure 1d) and the particle density at the glass substrate is varying monotonically with the concentration of the stock solution (Supplementary Figure 1e) demonstrating a well-controlled binding process. The laser power to heat conversion efficiency was calculated as the ratio of total power absorbed over the total power of the pump impinging on the sample surface. The total power absorbed was calculated by integrating the heat source density (HSD), as obtained from the temperature retrieval algorithm, over the whole surface. The plot in Supplementary Figure 1f shows how the light to heat conversion efficiency scales linearly with the surface particle density on the substrate. The power conversion efficiency from the HSD compares well with the maximum attenuation as measured from the glass spectra (Supplementary Figure 1g). Small deviations between these two measurements arise from inhomogeneity in the AuNR surface density and the fact that HSD-based power efficiency measures at the microscale, whereas the attenuation measures at the mm-scale.

We tuned the density of immobilized AuNRs on the substrate to be within 20-80 particles/μm^2 such that the dominant temperature distribution is determined by collective heating effects, whilst also minimizing optical coupling between the individual nanoparticles when the density is too high. In the collective heating regime, the temperature gradient is delocalized rather than confined to the proximity of each individual heat source, which it this case would be the AuNRs[6]. As a result of the dominant collective heating effect the overall temperature profile is smooth irrespective of the size of each individual source of heat.

Tracer particle characterization

To test the robustness of our 3D tracking we characterized the motion of the tracers during 1000 frames under equilibrium conditions using a mean-squared displacement, MSD, analysis.

The tracers were seeded in a flow cell with a nominal channel depth of 50 μm that was orientated perpendicular to the direction of gravity. The linear MSD curves, each with a slope proportional to the diffusion coefficient of the particle, confirmed that the particle dynamics correspond to Brownian motion (Supplementary Figure 2). From each slope we proceeded to calculate the size of each particle following the Stokes-Einstein equation, which states that the diffusion coefficient is inversely proportional to the hydrodynamic radius. The resulting particle size distribution, with a mean of 1.0 micron, agreed with the manufacturer's specifications, validating our approach to follow particle dynamics (Supplementary Figure 2 - inset).

We chose 1.0 μm polystyrene particles as tracers given the high signal to noise ratio associated with their localization, and because the size of the particles determines the number of samples required to effectively suppress the Brownian contribution[7]. Nonetheless, we could have also used smaller polystyrene particles, which move faster and thus require a higher number of samples to attain the same level of sensitivity without affecting the thermophoretic contribution[8].

Supplementary References

1. Mast, C. B., Schink, S., Gerland, U. & Braun, D. Escalation of polymerization in a thermal gradient. *Proc. Natl. Acad. Sci. U. S. A.* **110**, 8030–8035 (2013).
2. Keil, L. M. R., Möller, F. M., Kieß, M., Kudella, P. W. & Mast, C. B. Proton gradients and pH oscillations emerge from heat flow at the microscale. *Nat. Commun.* **8**, 1–9 (2017).
3. Duhr, S., Arduini, S. & Braun, D. Thermophoresis of DNA determined by microfluidic fluorescence. *Eur. Phys. J. E* **15**, 277–286 (2004).
4. Roxworthy, B. J., Bhuiya, A. M., Vanka, S. P. & Toussaint, K. C. Understanding and controlling plasmon-induced convection. *Nat. Commun.* **5**, 1–8 (2014).
5. Würger, A. Thermal non-equilibrium transport in colloids. *Reports Prog. Phys.* **73**, (2010).
6. Baffou, G. *et al.* Photoinduced heating of nanoparticle arrays. *ACS Nano* **7**, 6478–6488 (2013).
7. Matsuura, Y., Nakamura, A. & Kato, H. Nanoparticle tracking velocimetry by observing light scattering from individual particles. *Sensors Actuators, B Chem.* **256**, 1078–1085 (2018).
8. Braibanti, M., Vigolo, D. & Piazza, R. Does thermophoretic mobility depend on particle size? *Phys. Rev. Lett.* **100**, 1–4 (2008).
