# Supplementary material for: Long-range optofluidic control with plasmon heating
Source: Nat Commun. 2021 Mar 31;12:2001. doi: 10.1038/s41467-021-22280-3 (PMC8012589; doi:10.1038/s41467-021-22280-3)
Supplement: Supplementary file 3 — Description of Additional Supplementary Files [file 41467_2021_22280_MOESM3_ESM.pdf]

Description of Additional Supplementary Files

Supplementary Movie 1. Correlative 3D dynamics and temperature distribution for a sample oriented perpendicular to gravity and with a depth of 50 µm. Scale bar: 10 µm.

Supplementary Movie 2. Thermally driven particle depletion for a sample oriented perpendicular to gravity and with a depth of 50 µm. Scale bar: 10 µm.

Supplementary Movie 3. Correlative 3D dynamics and temperature distribution for a sample oriented parallel to gravity and with a depth of 50 µm. Scale bar: 10 µm.

Supplementary Movie 4. Competing transport phenomena at low sample depth for a sample oriented parallel to gravity and with a depth of 20 µm. Scale bar: 10 µm.

Supplementary Movie 5. Effect of different aqueous media on the fluid dynamics for a sample orientated parallel to gravity and with a depth of 100 µm. Scale bar: 10 µm.

Supplementary Movie 6. Evidence for hydrodynamic-based cell manipulation with a localized thermal gradient when the is sample orientated parallel to gravity and with a depth of 100 µm. Scale bar: 10 µm.

Supplementary Movie 7. Effect of temperature on the flow velocity (XZ projection). Scale bar: 10 µm.

Supplementary Movie 8. Effect of temperature on the flow velocity (YZ projection). Scale bar: 10 µm.

Supplementary Movie 9. Effect of chamber depth on the flow velocity (XZ projection). Scale bar: 10 µm.

Supplementary Movie 10. Effect of chamber depth on the flow velocity (YZ projection). Scale bar: 10 µm.

Supplementary Movie 11. Effect of heat source size on the flow velocity (XZ projection). Scale bar: 10 µm.

Supplementary Movie 12. Effect of heat source size on the flow velocity (YZ projection). Scale bar: 10 μm.

Supplementary Movie 13. Long-range effects probed by two different fields of view.

Supplementary Movie 14. Controlling the direction of flow in the proof-of-concept device by changing the location of the pump beam. Scale bar: 100 μm.

Supplementary Movie 15. Summary of the different dynamics observed in the proof-of-concept device. Scale bar: 100 μm.
